# Fast oxygen dynamics as a potential biomarker for epilepsy

**DOI:** 10.1038/s41598-018-36287-2

**Published:** 2018-12-18

**Authors:** Jordan S. Farrell, Quentin Greba, Terrance P. Snutch, John G. Howland, G. Campbell Teskey

**Affiliations:** 1 Hotchkiss Brain Institute, University of Calgary, Calgary, Canada; 2 Stanford University, Stanford, California USA; 3 University of Saskatchewan, Saskatoon, Canada; 4 Michael Smith Laboratories and Djavad Mowafaghian Centre for Brain Health, University of British Columbia, Vancouver, Canada

## Abstract

Changes in brain activity can entrain cerebrovascular dynamics, though this has not been extensively investigated in pathophysiology. We assessed whether pathological network activation (i.e. seizures) in the Genetic Absence Epilepsy Rat from Strasbourg (GAERS) could alter dynamic fluctuations in local oxygenation. Spontaneous absence seizures in an epileptic rat model robustly resulted in brief dips in cortical oxygenation and increased spectral oxygen power at frequencies greater than 0.08 Hz. Filtering oxygen data for these fast dynamics was sufficient to distinguish epileptic vs. non-epileptic rats. Furthermore, this approach distinguished brain regions with seizures from seizure-free brain regions in the epileptic rat strain. We suggest that fast oxygen dynamics may be a useful biomarker for seizure network identification and could be translated to commonly used clinical tools that measure cerebral hemodynamics.

## Introduction

Clinical tools to measure brain metabolism and blood flow are often used to identify abnormal changes in brain networksacross many disorders and diseases, including epilepsy, where a key goal is to identify the extent of the seizure-onset zone for potential surgical resection^1^. Analysis of these data generally provides a snapshot of a single time point (interictal, ictal, or postictal scans) and can be costly^2^ and of limited insight without combining other diagnostic resources^3–5^. A time-series analysis of oxygen dynamics (i.e. fluctuations on a short timescale) has not been investigated and could be a source of useful information regarding local network pathophysiology. In light of recent evidence indicating that oscillations in cerebrovascular dynamics are entrained with local field potential (LFP)^6^, we reasoned that pathophysiological changes in LFP (i.e. seizures) could perturb ongoing oxygen dynamics and serve as a potential biomarker.

## Results

**Figure 1:**
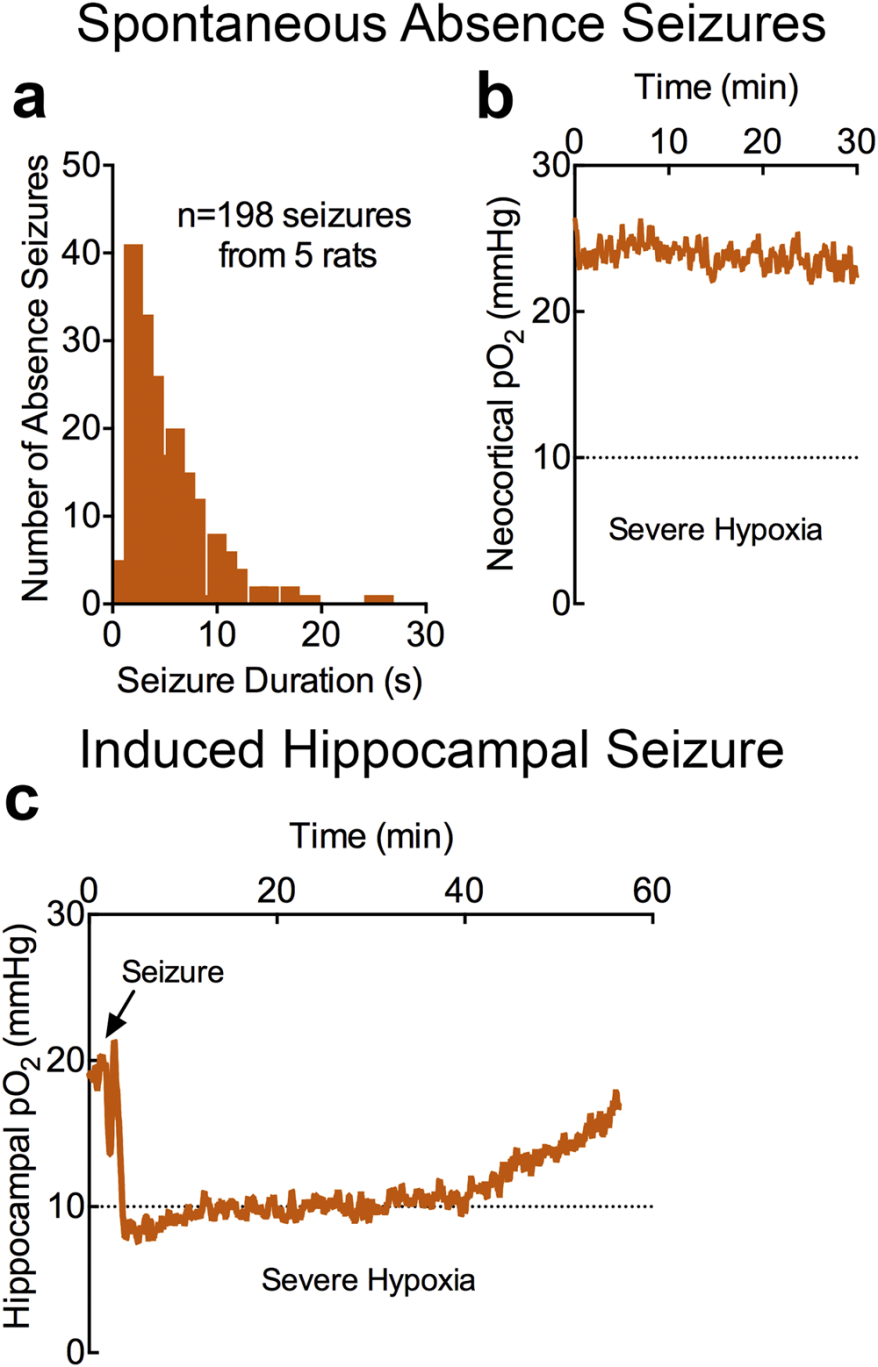
Absence seizures do not result in severe postictal hypoxia. (**a**) 198 seizures were recorded from 5 GAERS rats. Distribution of seizure durations from all seizure events. (**b**) Mean neocortical oxygen profile (n = 5) during 30-minute recording sessions. Mean oxygen did not fall below the severe hypoxic threshold, despite the occurrence of 198 absence seizures. (**c**), Mean hippocampal oxygen profile (n = 3) following elicitation of a single, brief seizure with 60 Hz kindling stimulation (first and only kindling session). Mean oxygen crossed the severe hypoxic threshold in the postictal period.

To address whether hemodynamic changes could be a useful epilepsy biomarker, we selected a rodent model of epilepsy with a high rate of spontaneous seizures. The Genetic Absence Epilepsy Rat from Strasbourg (GAERS) displays frequent corticothalamic seizures consistent with childhood absence epilepsy^7,8^. During 30-minute sessions, we recorded 198 seizures from 5 rats (Fig. 1a) that were never associated with severe postictal hypoxia (Fig. 1b). Since absence seizures were free of severe and long-lasting postictal hypoxia, which could be elicited by induced focal seizures (Fig. 1c)^9,10^, this is an ideal model to study oxygen dynamics associated with brief, hypersynchronous network events.

**Figure 2:**
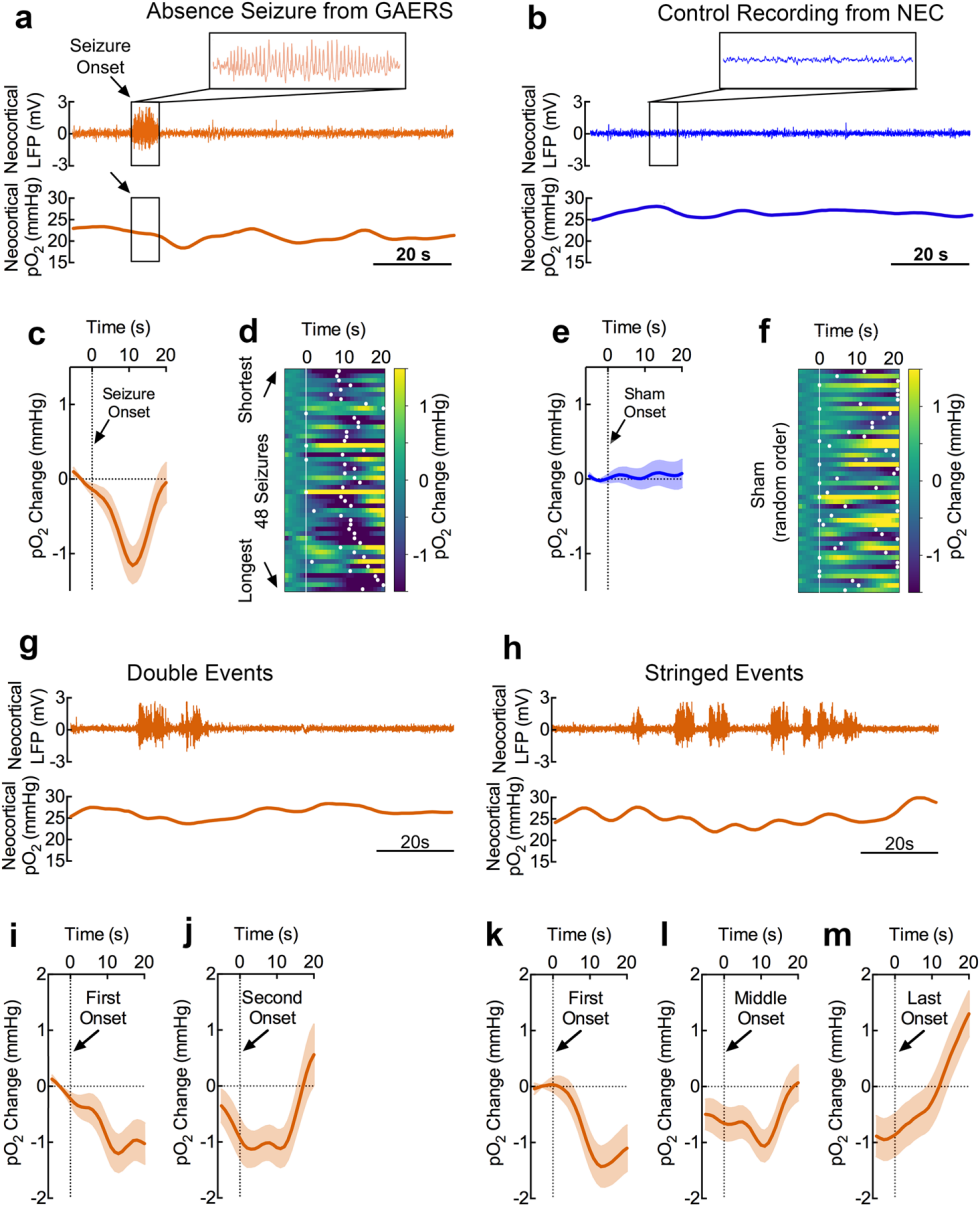
Absence seizures result in brief dips in neocortical oxygen which do not summate when repeated. (**a**) Neocortical LFP and pO_2_ recordings during a representative discrete seizure. Absence seizures were classified as discrete if another seizure was not observed within 20 s of seizure onset and termination. A brief dip in oxygen is observed ~10 s after seizure onset. Scale bar = 20 s. (**b**) Representative neocortical LFP and pO_2_ recordings from a seizure-free NEC rat. Scale bar = 20 s. (**c**) Mean neocortical pO_2_ during 48 discrete absence seizures. Profiles are aligned at seizure onset. Mean peak dip of 1.6 mmHg occurred at a mean of 10.6 s following seizure onset. (**d**) Relative spectrogram of neocortical pO_2_ organized from shortest to longest seizures in GAERS rats. Peak pO_2_ dip is visualized by white dots and is more delayed with longer seizures. (**e**) Mean neocortical pO_2_ during 48 sham events (randomly generated sham onsets, n = 48 from 3 NEC rats). (**f**) Relative spectrogram of NEC neocortical pO_2_ in random order. Peak pO_2_ dip is visualized by white dots. (**g**) Neocortical LFP and pO_2_ recordings during a representative “double” seizure event. Double events were defined when a second seizure began less than 20 s after the first seizure terminated and both events were isolated by a 20 s seizure-free gap before the first seizure began and after the second seizure terminated. A brief dip in oxygen is observed ~10 s after the first seizure onset and is blunted following the second seizure. Scale bar = 20 s. (**h**) Neocortical LFP and pO_2_ recordings during a representative “string” of seizures. Stringed events were defined by more than two seizures occurring without a > 20 s seizure-free gap after each preceding seizure. A brief dip in oxygen is observed ~10 s after the first seizure onset and subsequent events generate fast oxygen dynamics. Scale bar = 20 s. (**i**) Mean neocortical pO_2_ profile during double events aligned at first seizure onset (n = 24, from 4 GAERS rats). (**j**) Mean neocortical pO_2_ profile during double events aligned at second seizure onset (n = 24, from 4 GAERS rats). (**k**) Mean neocortical pO_2_ profile during stringed events aligned at first seizure onset (n = 25, from 5 GAERS rats). (**l**) Mean neocortical pO_2_ profile during stringed events aligned at middle seizure onset(s) (n = 52, from 5 GAERS rats). (**m**) Mean neocortical pO_2_ profile during stringed events aligned at last seizure onset (n = 25, from 5 GAERS rats).

We categorized seizures as discrete, double, or stringed events depending on whether a seizure was associated with another seizure within 20 seconds of seizure termination. Following discrete seizures (Fig. 2a), we observed brief dips in neocortical oxygen of 1.6 mmHg which peaked at 10.6 s following seizure onset (Fig. 2c). As expected, no seizures were observed in the non-epileptic control (NEC) strain (Fig. 2b) and no oxygen changes were observed at randomly chosen times (sham seizure onsets) (Fig. 2e). While longer seizures did not significantly alter the magnitude of this dip (Supplementary Fig. 1), they significantly delayed the peak oxygen dip (Fig. 2d vs. Fig. 2f, Supplementary Fig. 1). Notably, our results are consistent with previous clinical characterization of hemodynamics during absence seizures^11^, suggesting our recordings are clinically valid.

We then assessed whether double (Fig. 2g) or stringed seizures (Fig. 2h) would summate and drive oxygen to lower levels. In either case, repeated seizures did not increase the magnitude of the oxygen dip and revealed an oxygen overshoot following the final seizure (Fig. 2i–m), highlighting the brain’s ability to regulate oxygen. Importantly, oxygen inflections occurred ~10 s following each seizure onset and gave rise to fast oscillatory activity, clearly evident following a string of seizures (Fig. 2h).

**Figure 3:**
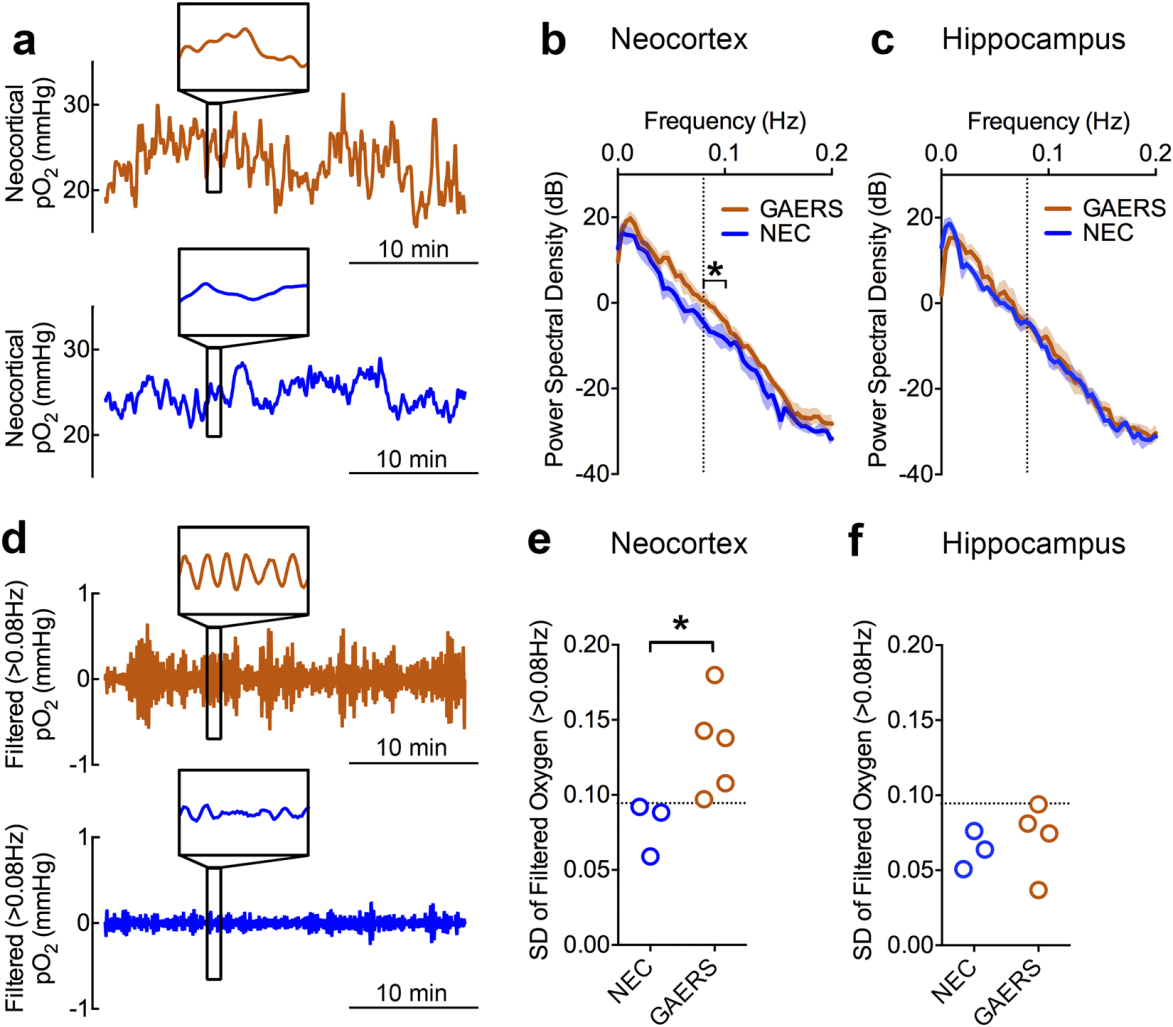
Fast oxygen dynamics are associated with absence seizures. (**a**) Representative neocortical pO_2_ recordings from a GAERS rat (orange) and NEC rat (blue). Inset shows 1 minute of data revealing fast oxygen dynamics overlaying slower oscillations. (**b**) Power spectral density analysis of GAERS (n = 5) and NEC (n = 3) oxygen recordings plotted on a logarithmic dB scale. Greater power in GAERs rats is observed between 0.08–0.1 Hz (t(6) = 3.19, *p < 0.05). (**c**) Analysis from (**b**) applied to hippocampal data (GAERS, n = 4; NEC, n = 3) revealing no significant change. (**d**) Representative filtered oxygen data (high-pass filter at 0.08 Hz) from Panel **a**. Inset shows 1 minute of data displaying enhanced amplitude at high frequency. (**e**,**f**) Standard deviation (SD) of filtered oxygen data for neocortical and hippocampal recordings, respectively. GAERS rats have significantly higher neocortical SD than NEC controls (t(6) = 2.55, *p < 0.05). A line drawn at an SD of 0.095 mmHg is able to separate epileptic rats from controls and distinguish non-epileptic brain regions.

**Figure 4:**
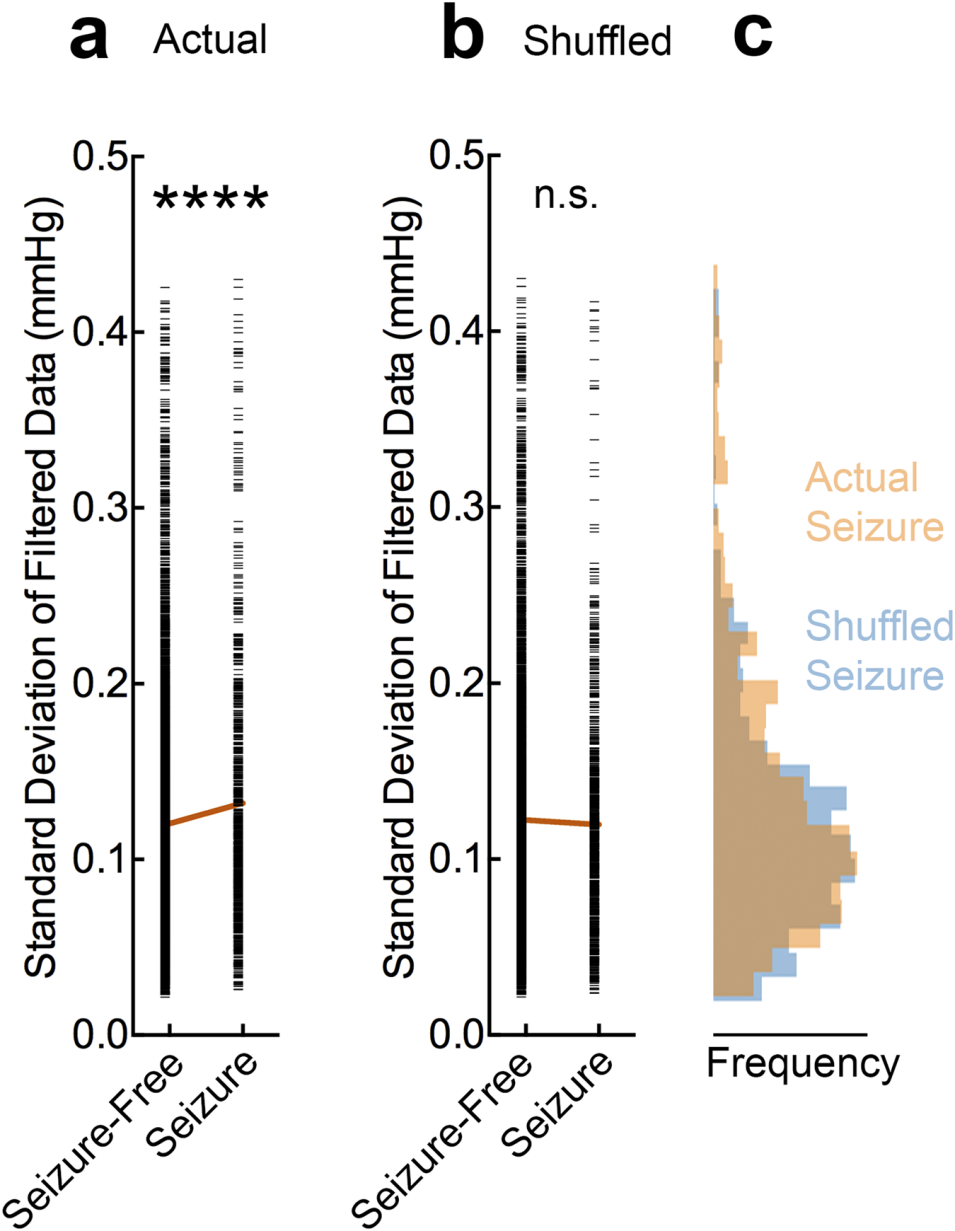
Seizure activity is correlated with fast oxygen dynamics. (**a**) Oxygen data was correlated with seizure-free or seizure periods as defined by LFP. A sliding window of 20 s was used to calculated the standard deviation of filtered data (>0.08 Hz) and aligned with the preceding 20 s of LFP to account for the lag in oxygen response. These two variables were significantly correlated (Linear Regression, R^2^ = 0.003, F = 29, ****p < 0.0001). (**b**) Data were shuffled as a control to misalign the two variables. No correlation was observed (Linear Regression, R^2^ = 0.0002, F = 1.5, p = 0.22). (**c**) Corresponding histograms to seizure data in (**a**,**b**). Overlap in the distributions is represented by brown (i.e. where the two colors mix).

Bolstered by this observation, we postulated that the presence of absence seizures could be determined solely based on the spectral characteristics of oxygen data. Prior research determined that cerebrovascular dynamics generally fluctuate at frequencies less than 0.1 Hz^6^. Given the fast kinetics of oxygen dips, especially during stringed events, seizures could increase the amplitude of high frequency oxygen changes. We performed power spectral density analysis on 30-minute recordings from GAERS and NEC rats (Fig. 3a) and, indeed, observed significantly increased power between 0.08–0.1 Hz (Fig. 3b). Since these changes were not observed at a control site without seizures (hippocampus) (Fig. 3c) and are correlated with the occurrence of seizures (Fig. 4a,b), fast oxygen dynamics reflect pathological network activity.

To further isolate the increase in fast oxygen dynamics, we applied a digital high-pass filter to the data to observe only the oscillatory components at these high frequencies (Fig. 3d). By simply measuring the standard deviation of the filtered data, we could distinguish between epileptic vs. non-epileptic rat strains (Fig. 3e). Moreover, in a brain region without seizures (hippocampus) in the epileptic rat strain, none of the data points crossed the threshold to be classified as epileptic (Fig. 3f). While this measurement was associated with the occurrence of seizures (Fig. 4a,b), these oxygen characteristics were not strictly unique to the epileptic brain since the control strain also had oscillations at this frequency, albeit markedly reduced (Fig. 3b). Rather, seizures shifted the distribution of high-frequency oxygen oscillations to higher amplitudes in the GAERS rats (Fig. 4c). Therefore, this analysis is suitable for identifying active epileptic networks, rather than the occurrence of individual seizures.

**Figure 5:**
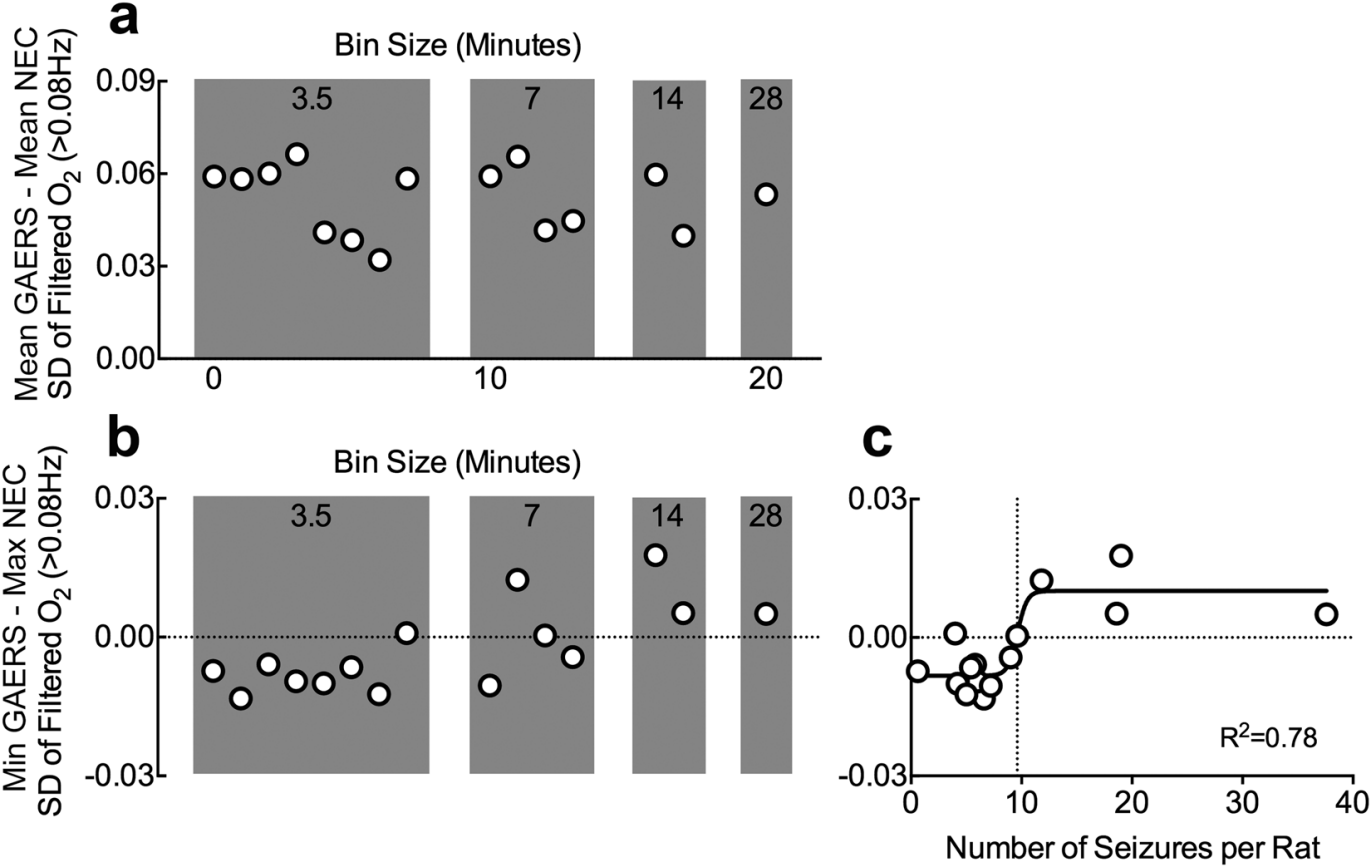
Minimum recording duration to categorize data as epileptic vs. control is between 7 and 14 minutes or about 10 seizures. Neocortical oxygen data was binned as displayed and filtered for fast oxygen dynamics (>0.08 Hz). We computed the differences in group means (**a**) or between the lowest point in the GAERS group from the highest point in the NEC group (**b**), such that a positive value in (**b)** would indicate that there is no overlap between groups. (**a**) Binning data into shorter epochs did not dramatically alter the differences in group means. (**b**) Binning data into shorter epochs resulted in the appearance to negative values, indicating that the group distributions started to overlap (i.e. highest value in the NEC group was greater than the lowest of the GAERS group). (**c**) Data from (**b)** plotted against the corresponding number of seizures (mean across rats) for that bin and fitted to a sigmoidal curve. The curve crossed from negative to positive values at 9.6 seizures per rat.

Lastly, we assessed the ability of this analysis to separate data into epileptic vs. control during smaller sampling intervals. Recordings of 7 minutes or less were associated with reduced accuracy, but separation was maintained with 14 minute bins or a minimum of 10 seizures per rat on average (Fig. 5a–c). Thus, we have identified fast oxygen dynamics as a potential biomarker of absence epilepsy and is sufficient to distinguish active epileptic brain networks from non-epileptic within 14 minutes of recording.

## Discussion

We first showed that absence seizures were associated with brief, mild dips in cortical oxygenation within the normoxic range, but importantly, we never observed severe postictal hypoxia (<10 mmHg). However, severe postictal hypoxia lasting approximately an hour was induced by modelling focal seizures with kindling stimulation. This provides evidence that GAERS rats possess the neurovascular mechanisms that orchestrate this pathological event, but that spontaneous corticothalamic seizures are fundamentally different. Since absence seizures are widely considered to not be associated with a true postictal state and are also free of postictal stroke-like events, severe postictal hypoperfusion/hypoxia could be the physiological driver underlying the postictal state. Moreover, given the potential negative impact of repeated postictal stroke-like events to the pathophysiology of epilepsy^12^, this may also explain the relative severity of different clinical seizure types and why absence seizures are relatively mild.

We then demonstrated that isolation of fast oxygen dynamics was sufficient to identify epileptic networks even though the dips in cortical oxygenation were relatively small. Therefore, this simple analytic approach may be a highly-sensitive biomarker for epilepsy. Moreover, the simplicity of this observation and availability of tools to study cerebrovascular dynamics makes this analysis adaptable to other datasets. Clinically, techniques such as functional magnetic resonance imaging (fMRI) or near infrared spectroscopy (NIRS) are suitable candidates. The only requirement for these measurements is a sufficiently high sampling rate (minimum of 0.2 Hz) and appropriate controls. As we have shown, brain regions without seizures can serve as a potential within-subject control.

It is also possible that pathological network activity in other epilepsies, independent of overt seizures, could also result in similar deviations in oxygen spectral characteristics. Interictal spikes (IIS) and pathological high-frequency oscillations (HFOs or fast ripples) have been identified as biomarkers of epileptogenic tissue^13–15^, but often require implantation of depth electrodes, which is both invasive and costly. IISs, for example, display BOLD-related changes^16^ and may be sufficient to perturb oxygen dynamics. Thus, this analytic approach could extend to other types of pathological epileptic activity and be captured by non-invasive imaging techniques. Thus, we have identified fast oxygen dynamics as a potential diagnostic biomarker for epileptiform events and this promising discovery opens the door to further characterize pathological cerebrovascular dynamics.

## Methods

### Rats

All recordings we performed on four-month-old GAERS and NEC rats (205–265 g) under awake, freely-moving conditions. Rats were bred and housed at the University of Saskatchewan as previously described^17^. Experiments were approved by the UofS Animal Research Ethics Board in accordance with the Canadian Council on Animal Care Guidelines.

### Oxygen and LFP recordings

Local tissue oxygenation was measured through chronically-implanted optrodes (Oxford Optronix) as previously described^9^. Bipolar electrodes for differential LFP recordings were constructed from insulated nichrome wire with a tip separation of ~1 mm (inVivo1). The following coordinates relative to bregma were used: Neocortex optrode (3.0 mm lateral, 2.0 mm ventral), neocortex electrode (1.0 mm anterior, 1.5 mm lateral, 3.0 mm ventral), hippocampus optrode (3.5 mm posterior, 3.5 mm lateral, 4.0 mm ventral) hippocampus electrode (3.0 mm posterior, 0.5 mm lateral, 4.0 mm ventral). Implants were secured with 3.2 mm stainless steel screws (one served as ground) and dental cement. Wideband LFP signals were amplified 1000X-2000X and digitized at 100 Hz (Grass Technologies). Oxygen data was sampled and digitized at 1 Hz. Prior to recording sessions, rats were extensively handled and then habituated to the recording chamber for 1–2 sessions. All recordings were done during the light cycle. Control and epileptic recordings were alternated to control for potential circadian effects.

### Analysis

Python 3.6 and GraphPad Prism 6 were used for analysis and figure generation. Figures were compiled in Adobe Photoshop. Seizures were categorized as outlined in the main text and oxygen data was aligned to seizure onset to observe seizure-induced oxygen changes. To isolate the relative changes and remove the absolute component of the data, a sliding average of 60 s was subtracted from the data. 1 minute of data was removed on both ends of the data. This manipulation enabled us to do power spectral density analysis (using Matplotlib in Python 3.6) and made the data more comparable to techniques only capable of measuring relative changes, which are most commonly used. To filter the data for high frequency components, a 5^th^ order high-pass Butterworth filter was constructed in Python 3.6 using Signal in SciPy with a 0.08 Hz cut-off. All statistical analyses were performed in Prism 6 and reported in figure captions.

## Electronic supplementary material

Farrell et al Supplementary Info

## Data Availability

The datasets and code generated during and/or analysed during the current study are available from the corresponding author on reasonable request.
