# Supplementary material for: Fast oxygen dynamics as a potential biomarker for epilepsy
Source: Sci Rep. 2018 Dec 18;8:17935. doi: 10.1038/s41598-018-36287-2 (PMC6298949; doi:10.1038/s41598-018-36287-2)
Supplement: Supplementary file 1 — Farrell et al Supplementary Info [file 41598_2018_36287_MOESM1_ESM.docx]

**Fast oxygen dynamics as a potential biomarker for epilepsy**

Jordan S. Farrell^1,2^, Quentin Greba^3^, Terrance P. Snutch^4^ John G. Howland*^3^, G. Campbell Teskey*^1^

**Supplementary Data**


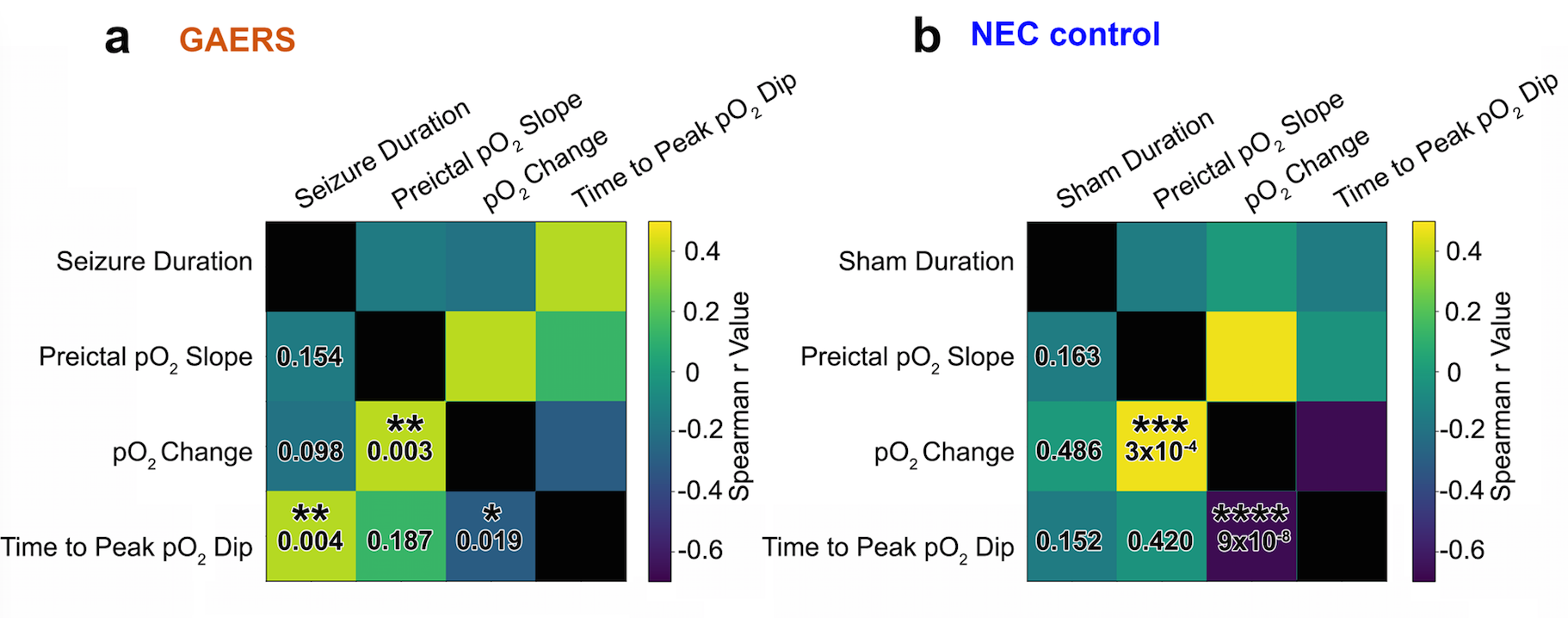


**Supplementary Fig. 1 – Factors affecting timing and severity of oxygen dips**

**a**, Correlations between variables potentially affecting dips in neocortical oxygen following discrete absence seizures (n=48 discrete events from 5 GAERS rats). Spearman r values are visualized by color (see scale). **b**, Correlations between variables potentially affecting dips in neocortical oxygen following sham events (n=48 events from 3 NEC rats). Spearman r values are visualized by color (see scale). *p<0.05, **p<0.01, ***p<0.001, ****p<0.0001.
